# 'Aspirin resistance' or treatment non-compliance: Which is to blame for cardiovascular complications?

**DOI:** 10.1186/1479-5876-6-47

**Published:** 2008-08-29

**Authors:** Eduard Shantsila, Gregory YH Lip

**Affiliations:** 1 Haemostasis Thrombosis and Vascular Biology Unit, University Department of Medicine, City Hospital, Birmingham, UK

## Abstract

Aspirin is one of the 'cornerstone' drugs in our current management of cardiovascular disorders. However, despite the prescription of aspirin recurrent vascular events still occur in 10–20% of patients. These, data together with the observations of diminished antiaggregatory response to aspirin in some subjects have provided the basis of the current debate on the existence of so-called "aspirin resistance". Unfortunately, many of the tests employed to define 'aspirin resistance' lack sufficient sensitivity, specificity, and reproducibility. The prevalence of 'aspirin resistance' as defined by each test varies widely, and furthermore, the value of a single point estimate measure of aspirin resistance is questionable. The rate of 'aspirin resistance' is law if patients observed to ingest aspirin, with large proportion of patients to be pseudo-'aspirin resistant', due to non-compliance. What are the implications for clinical practice? Possible non-adherence to aspirin prescription should also be carefully considered before changing to higher aspirin doses, other antiplatelet drugs (e.g. clopidogrel) or even combination antiplatelet drug therapy. Given the multifactorial nature of atherothrombotic disease, it is not surprising that only about 25% of all cardiovascular complications can usually be prevented by any single medication. We would advocate against routine testing of platelet sensitivity to aspirin (as an attempt to look for 'aspirin resistance') but rather, to highlight the importance of clinicians and public attention to the problem of treatment non-compliance.

## Editorial

Aspirin is one of the 'cornerstone' drugs in our current management of cardiovascular disorders. The metaanalysis from the Antithrombotic Trialists' Collaboration of 287 randomized trials of antiplatelet therapy in patients at high risk of occlusive vascular events demonstrated a 32% reduction in nonfatal myocardial infarction (MI), nonfatal stroke, and vascular death in patients treated with aspirin [1]. However, despite the prescription of aspirin recurrent vascular events still occur in 10–20% of patients [2]. These, data together with the observations of diminished antiaggregatory response to aspirin in some subjects have provided the basis of the current debate on the existence of so-called "aspirin resistance".

'Aspirin resistance' has been defined as either the failure of aspirin to fully inhibit platelet aggregation in the laboratory setting or (clinically) as its inability to prevent cardiovascular events. The problem is important as it potentially implies the need for repeated laboratory tests and/or the replacement of aspirin by other antiplatelet drugs in millions of patients [3].

Unfortunately, many of the tests employed to define 'aspirin resistance' lack sufficient sensitivity, specificity, and reproducibility [4]. The prevalence of 'aspirin resistance' as defined by each test varies widely, and furthermore, the value of a single point estimate measure of aspirin resistance is questionable [4,5]. Indeed, the insufficient laboratory suppression of platelet activity may result from reduced enteral absorption of aspirin (e.g. when low doses of enteric-coated aspirin are used), the concomitant administration of other cyclooxygenase-1 inhibitors (e.g. ibuprofen and naproxen), or even increased platelet turnover (e.g. as in infection, inflammation and following major surgery) [6-9]. Polymorphism of genes involved in the thromboxane biosynthetic pathway may also be associated with a modification of the response to aspirin and its clinical efficacy [10]. Partial loss of antiplatelet effect during long-term aspirin treatment has also been suggested [11,12]. Of note, monocytes/macrophages produce large amounts of thromboxane synthase making them platelet-independent source of thromboxane A2 generation, even when platelet activity is effectively suppressed [13].

When a stroke or myocardial infarction occurs in a patient on aspirin therapy, it is unknown if the patient was taking the prescribed aspirin as prescribed prior to the event. Sadly, up to 40% of patients with cardiovascular disease do not comply with aspirin [14-17]. Thus, poor compliance may be an important reason why aspirin is ineffective in the laboratory and clinically settings. It is worth emphasizing that a lack of drug compliance is characteristic for many chronic treatments and aspirin is not exception. The relatively high rate of gastrointestinal complications with aspirin (and patients' awareness of such symptoms) often make aspirin 'first-choice-to-stop' drug from an often long list of prescribed treatments (antihypertensives, lipid lowering drugs, antianginals, etc.) in patients with cardiovascular disease. Failure to follow aspirin prescription may be greater in patients with co-morbidities (e.g. in older patients) [18]. Post-MI patients with low educational status (e.g. not graduating from high school) are also more likely to discontinue use of all medications; the same applies for older patients, especially women [19].

A recent multicenter prospective cohort, the Prospective Registry Evaluating Myocardial Infarction: Event and Recovery (PREMIER) study, has demonstrated shocking data on non-adherence to medications in patients after acute MI [18]. At 1 month after hospital discharge with a prescription of aspirin, beta-blockers and statins, 12% of patients discontinued use of all 3 medications, whilst 4% discontinued use of 2 medications and 18% discontinued use of 1 drug. The patients who completely stopped their drug treatments had lower 1-year survival (88.5% vs. 97.7%) compared to those who continued to take at least one medication [18].

Another published systematic review and meta-analysis on the possible relation between 'aspirin resistance' and clinical outcomes in patients with cardiovascular disease (20 studies, 2930 patients) reported that 28% of patients can be classified as 'aspirin resistant' [20]. The latter was associated with a sharp increase in the rate of cardiovascular related events (41% of aspirin resistant patients, with odd ratio [OR] 3.85), death (5.7%, OR 5.99) and recurrent acute coronary syndromes (39.4%, OR 4.06). Clearly, the implications are grave.

What may be an especially important observation is that these 'aspirin resistant' patients usually did not benefit from other antiplatelet treatments. Does it indicate the presence of multidrug platelet resistance? Alternatively, should patients' should be questioned? The authors of the meta-analysis state that compliance was assessed by the primary study investigator in only 17 of the 20 studies included in analysis, and this was by telephone or interviews, or more directly by the patient's presence in hospital [20]. Nonetheless, few of the studies confirmed compliance by measurement of a biochemical marker of compliance. Of note odd ratios of cardiovascular events in 'aspirin resistant' patients in this meta-analysis were very close to the hazard ratio (3.81) in those who discontinued all medications (including aspirin) in PREMIER study [18,20].

Are patient interview data on drug adherence sufficiently reliable? Tantry et al. found that among 223 patients with coronary artery disease who were reported to use aspirin regularly, only 8 patients had long-term 'aspirin resistance' as estimated by arachidonic acid-induced light aggregation and thrombelastography [21]. However, 7 of these 8 patients admitted to being non-compliant on repeated interviews and all became 'aspirin sensitive' after in-hospital aspirin administration, and only 1 patient (~0.4%) was truly resistant to aspirin treatment.

Cotter et al. measured thromboxane B2 production in 73 acute MI survivors and found that thromboxane production was not suppressed in 21 patients (29%), indicating some degree of 'aspirin resistance' [22]. When questioned, 12 of the 21 admitted that they were not taking aspirin as recommended. The clinical impact of aspirin non-compliance over supposed 'aspirin resistance' in MI survivors was demonstrated by the fact that patients who admitted poor drug compliance had substantially higher rates of cardiovascular events (42%) and readmissions (67%) when compared those with lack of response to aspirin administration (11% for both end points) [22]. Of note, 'aspirin resistance' in this laboratory study did not seem to affect prognosis significantly when compared to adherent responders, with 6% of these suffering recurrent cardiovascular events and 11% had re-admissions to hospital [22].

In fact aspirin non-adherence is associated with the presence of anginal symptoms. For example, Carney et al. provided patients with coronary artery disease with an aspirin packaged equipped with an electronic adherence monitor, and found that symptomatic patients took aspirin on 62.4% of the days, compared to 77.3% of days in the patients without symptoms [14]. These data indicate a high rate of aspirin not-compliance even in those who suffer symptoms (angina) [14]. Tarján et al. revealed that in patients with acute MI and unstable angina 'aspirin resistance' [laboratory-defined] was seen in 34% of cases, and almost third of them did not have any traces of aspirin metabolites in their urine [15]. We can only speculate on the possible rate of aspirin non-compliance when this drug is prescribed for primary prevention.

In the current issue of the *Journal of Translational Medicine*, Schwartz et al. show that in a population of post MI patients observed to ingest aspirin and whose platelets studied 2 hours post ingestion had decreased light aggregation response to arachidonic acid [23]. Only a small percentage of these patients (3.4%) could be classified as resistant. A large proportion of patients (8.4%) was found to be pseudo-'aspirin resistant', due to non-compliance and were reclassified as 'normal responders' following compliant aspirin uptake. No significant difference in aspirin antiplatelet activity was found between normal subjects and post-MI patients indicating the factors other than long-term aspirin administration per se may be involved in the reduced response to aspirin [23]. These data provide additional and persuasive evidence that undetected non-compliance may lead to an overestimate of the rate of 'aspirin resistance' [19]. Furthermore, Schwartz et al. [23] introduce a novel index for measuring aspirin's platelet inhibitory effect, the net aspirin response. The net aspirin response measures the amount of aspirin induced platelet inhibition by subtracting the aggregation response on aspirin from the aggregation response off aspirin state and was shown to be statistically normally distributed. Platelets from patients with a decreased net aspirin response may for unknown reasons be relatively less dependent on the arachidonic acid pathway for activation. Indeed, the paper by Schwartz et al. may be additional basis to question the reliability of 'interview proven compliance' and advocate mandatory laboratory evaluation for aspirin's platelet inhibitory effect in future studies on 'aspirin resistance' [23].

What are the implications for clinical practice? Possible non-adherence to aspirin prescription should also be carefully considered before changing to higher aspirin doses, other antiplatelet drugs (e.g. clopidogrel) or even combination antiplatelet drug therapy [24]. Given the multifactorial nature of atherothrombotic disease, it is not surprising that only about 25% of all cardiovascular complications can usually be prevented by any single medication [25]. We would advocate against routine testing of platelet sensitivity to aspirin (as an attempt to look for 'aspirin resistance') but rather, to highlight the importance of clinicians and public attention to the problem of treatment non-compliance.

## Competing interests

ES declares that he has no competing interests.

GYHL was clinical adviser to the guideline development group writing the NICE Guidelines on AF management. He has received funding for research, educational symposia, consultancy and lecturing from different manufacturers of drugs used for the treatment of thrombosis.

## Authors' contributions

Both ES and GYHL carried out the manuscript preparation. All authors read and approved the final manuscript.
